# Apolipoprotein C-III is linked to the insulin resistance and beta-cell dysfunction that are present in rheumatoid arthritis

**DOI:** 10.1186/s13075-022-02822-w

**Published:** 2022-05-30

**Authors:** Candelaria Martín-González, Tomás Martín-Folgueras, Juan Carlos Quevedo-Abeledo, Antonia de Vera-González, Alejandra González-Delgado, Laura de Armas-Rillo, Miguel Á. González-Gay, Iván Ferraz-Amaro

**Affiliations:** 1 Division of Internal Medicine, Hospital Universitario de Canarias, Tenerife, Spain; 2 Department of Internal Medicine, University of La Laguna (ULL), Tenerife, Spain; 3 Division of Endocrinology, Hospital Universitario de Canarias, Tenerife, Spain; 4 Division of Rheumatology, Hospital Doctor Negrín, Las Palmas de Gran Canaria, Spain; 5 Division of Central Laboratory, Hospital Universitario de Canarias, Tenerife, Spain; 6 Division of Health Sciences, Universidad Europea de Canarias, Tenerife, Spain; 7 Epidemiology, Genetics and Atherosclerosis Research Group On Systemic Inflammatory Diseases, Hospital Universitario Marqués de Valdecilla, IDIVAL, Santander, Spain; 8 Division of Rheumatology, Hospital Universitario Marqués de Valdecilla, Universidad de Cantabria, Santander, Spain; 9 Cardiovascular Pathophysiology and Genomics Research Unit, School of Physiology, Faculty of Health Sciences, University of the Witwatersrand, Johannesburg, South Africa; 10 Division of Rheumatology, Hospital Universitario de Canarias, Tenerife, Spain

**Keywords:** Rheumatoid arthritis, Insulin resistance, Beta-cell dysfunction, Apolipoprotein C-III

## Abstract

**Background:**

Insulin resistance and beta-cell dysfunction are manifestations of rheumatoid arthritis (RA). Apolipoprotein C-III (ApoC3) has been associated with such insulin resistance and beta-cell dysfunction in the general population. Our purpose was to study whether ApoC3 is also related to the insulin resistance and beta-cell dysfunction that are present in patients with RA.

**Methods:**

Three hundred thirty-eight non-diabetic patients with RA who had a glycemia lower than 110 mg/dl were recruited. Insulin, C-peptide, and ApoC3 were assessed. Insulin resistance and beta-cell function were calculated using the Homeostasis Model Assessment (HOMA2) indices. A multivariable regression analysis was performed to study the relationship of ApoC3 with those molecules and indices adjusting for classic factors associated with insulin resistance that included glucocorticoids.

**Results:**

ApoC3 was related to significant higher levels of circulating insulin (beta coef. 0.37 [95%CI 0.01–0.73] µU/ml, *p* = 0.044) and C-peptide (beta coef. 0.13 [95%CI 0.05–0.22] ng/ml, *p* = 0.003), and higher insulin resistance —HOMA2-IR— (beta coef. 0.05 [95%CI 0.00–0.09], *p* = 0.041) and beta-cell dysfunction —HOMA2-%B— (beta coef. 2.94 [95%CI 0.07–5.80], *p* = 0.044) indices. This was found after a fully multivariable analysis that included, among others, prednisone intake and the classic factors associated with carbohydrate metabolism such as triglycerides, waist circumference, and obesity.

**Conclusion:**

ApoC3, insulin resistance, and beta-cell dysfunction are independently associated in patients RA.

## Background

Insulin resistance (IR) is defined as the inability of a known quantity of insulin to increase glucose uptake and utilization in an individual as much as it does in a normal population [1]. As IR worsens, the resulting hyperglycemia leads to beta-cell dysfunction that is initially characterized by hypersecretion of insulin and C-peptide, but that afterwards leads to a defect in the secretion of both molecules as the beta cell eventually fails [2]. Therefore, beta-cell dysfunction and IR are interrelated processes but they represent different pathophysiological phenomena. Both pathological states influence each other and presumably synergistically are critical precursors of type 2 diabetes [3] and have been linked with cardiovascular disease [4].

There is sufficient evidence regarding the fact that rheumatoid arthritis (RA) is associated with both beta-cell dysfunction and IR [5–10]. This is believed to be due to the systemic inflammation that accompanies the disease that is capable of causing a disruption in the carbohydrate metabolism. However, beyond defining its presence, there are no studies that establish the exact mechanisms responsible for IR and beta-cell dysfunction in patients with RA.

Apolipoprotein C-III (ApoC3) is a regulator of triglyceride-rich lipoproteins in the circulation. It has been identified as an inhibitor of lipoprotein lipase activity and a key regulator of triglycerides concentrations in plasma. Because loss-of-function mutations in ApoC3 are characterized by a strong decrease in serum triglycerides and decreased cardiovascular risk, ApoC3 has been suggested to be a potent therapeutic approach to control dyslipidemia and cardiovascular disease [11]. ApoC3 has also been shown to serve as a link between IR and beta-cell failure. The mechanistic explanation is that specific IR within the pancreatic islet leads to local expression of ApoC3, resulting in an autocrine negative feedback loop for beta-cell function and survival [12]. Besides, ApoC3 enhances pancreatic beta-cell apoptosis via an increase of the cytoplasmic Ca2 + levels in the insulin-producing cells. In addition, overexpression of ApoC3 augments non-alcoholic fatty liver disease and exacerbates inflammatory pathways in skeletal muscles, affecting insulin signaling and thereby inducing IR. Moreover, recent studies reveal a possible mechanism of body weight increase and glucose production through a potential ApoC3-induced lipoprotein lipase inhibition in the hypothalamus. Also, the presence of ApoC3 on the surface of high-density lipoprotein particles is associated with impairment of their antiglycemic and atheroprotective properties [13].

In the present work, our objective was to study, in a large series of RA patients, whether ApoC3 is associated with both IR and beta-cell dysfunction that are present in RA patients. If so, it would imply that this molecule would be part of the pathophysiological pathways involved in the occurrence of both processes in patients with RA.

## Material and methods

### Study participants

This was a cross-sectional study that included 338 patients with RA. All of them were 18 years old or older and fulfilled the 2010 ACR/EULAR diagnostic criteria [14]. They had been diagnosed by rheumatologists and were periodically followed-up at Rheumatology outpatient clinics. For the purposes of inclusion in the present study, the duration of RA disease was required to be ≥ 1 year. Patients with diabetes mellitus were excluded. Furthermore, all patients had a glycemia < 110 mg/dl, and none of them were on glucose-lowering drugs or insulin therapy. Patients taking prednisone ≤ 10 mg /day or an equivalent dose were not excluded, as glucocorticoids are often used in the treatment of RA. Patients were excluded if they had a history of myocardial infarction, angina, stroke, a glomerular filtration rate < 60 ml/min/1.73 m^2^, a history of cancer, or any other chronic disease, or evidence of active infection. The study protocol was approved by the Institutional Review Committee at Hospital Universitario de Canarias and at Hospital Universitario Doctor Negrín (both in Spain), and all subjects provided informed written consent (approval no. 2015_84).

### Data collection and laboratory assessments

Individuals included in the study completed a CV risk factor and medication use questionnaire and underwent a physical examination. Body-mass index (the weight in kilograms divided by the square of the height in meters), abdominal circumference, and systolic and diastolic blood pressure were assessed under standardized conditions. Information regarding smoking status and hypertension was obtained from the questionnaire. Medical records were reviewed to ascertain specific diagnoses and medications. Disease activity in patients with RA was measured using the Disease Activity Score (DAS28) in 28 joints [15], the Clinical Disease Activity Index (CDAI) [16], and the Simple Disease Activity Index (SDAI) [17].

The homeostatic model assessment (HOMA) method was performed to determine IR. Briefly, the HOMA model enabled an estimate of insulin sensitivity (%S) and β-cell function (%B) from fasting plasma insulin, C peptide, and glucose concentrations. In this study, we used HOMA2, the updated-computer HOMA model [18]. HOMA2 has nonlinear solutions and this updated model (compared to the minimal model HOMA) accounts for variations in hepatic and peripheral glucose resistance (i.e., the reduction in the suppression of hepatic glucose output by hyperglycemia and the reduction of peripheral glucose-stimulated glucose uptake). In HOMA2, the insulin secretion curve has been modified to allow for an increase in insulin secretion in response to a plasma glucose concentration of 10 mmol/l. This model can be used to assess not only insulin sensitivity but beta-cell function from paired fasting plasma glucose and specific insulin, or C peptide, concentrations across a range of 1–2200 pmol/l for insulin and 1–25 mmol/l for glucose. Since C peptide is a marker of secretion, this is used for the estimation of β-cell function; and insulin data is preferable when calculating %S since HOMA-%S is derived from glucose disposal as a function of insulin concentration. However, to better express both IR and beta-cell function, in our study, IR, %S, and %B were calculated using equally insulin and C-peptide serum levels. The computer model provided a value for insulin sensitivity expressed as HOMA2-%S (in which 100% is normal). HOMA2-IR (insulin resistance index) is simply the reciprocal of %S. For the detection of ApoC3, an ELISA kit was used (Elabscience, USA). No significant cross-reactivity or interference between human ApoC3 and analogs is observed with this kit. Both intra and inter-coefficients of variability are < 10% for this assay. Cholesterol, triglycerides, and HDL cholesterol were measured using the enzymatic colorimetric assay. LDL cholesterol was calculated using the Friedewald formula.

### Statistical analysis

Demographic and clinical characteristics in patients with RA were described as mean (standard deviation) or percentages for categorical variables. For non-normally distributed continuous variables, data were expressed as median and interquartile range (IQR). Multivariable linear regression analysis, adjusting for confounders, was assessed to analyze the association between ApoC3 and glucose homeostasis molecules and indexes. Confounding variables in the relation of ApoC3 to IR indexes were selected from those variables that had a univariable relation to ApoC3 with a *p* value inferior to 0.20. Beta coefficients of the linear regression models are shown unstandardized. All the analyses used a 5% two-sided significance level and were performed using Stata software, version 17/SE (StataCorp, College Station, TX, USA). *P* values < 0.05 were considered statistically significant.

## Results

### Demographic and disease-related data

A total of 338 patients with RA were included in this study. Demographic- and disease-related characteristics of the participants are shown in Table 1. The mean age was 54 ± 10 years and 84% of the patients were women. Patients had a body mass index and an abdominal circumference of 29 ± 17 kg/m^2^ and 96 ± 13 cm, respectively. Traditional CV risk factors were commonly observed. In this regard, 22% were current smokers, 29% had a body mass index equal or higher than 30 kg/m^2^, and hypertension was present in the 30% of the patients. Besides, 28% of them were taking statins at the time of the study. The full lipid profile is described in Table 1.

**Table 1:** Demographics, cardiovascular risk factors, and disease-related data in patients with RA

	RA
	(*n* = 338)
Age, years	54 ± 10
Female, *n* (%)	284 (84)
BMI, kg/m^2^	29 ± 17
Abdominal circumference, cm	96 ± 13
Cardiovascular data	
CV risk factors, *n* (%)	
Current smoker	75 (22)
Obesity	98 (29)
Hypertension	100 (30)
Diabetes mellitus	–
Statins, *n* (%)	95 (28)
Lipids	
Total cholesterol, mg/dl	207 ± 37
Triglycerides, mg/dl	138 ± 78
HDL cholesterol, mg/dl	57 ± 15
LDL cholesterol, mg/dl	122 ± 32
LDL:HDL cholesterol ratio	2.29 ± 0.90
Non-HDL cholesterol, mg/dl	150 ± 37
Lipoprotein (a), mg/dl	35 (12–102)
Apolipoprotein A1, mg/dl	173 ± 30
Apolipoprotein B, mg/dl	108 ± 46
Apo B:Apo A ratio	0.64 ± 0.24
Apolipoprotein C-III, mg/dl	4.6 (2.0–8.4)
Disease-related data	
Disease duration, years	8 (4–15)
CRP at time of study, mg/l	2.6 (1.2–5.6)
ESR at time of study, mm/1º h	18 (7–32)
Rheumatoid factor, *n* (%)	250 (75)
ACPA, *n* (%)	204 (62)
DAS28-ESR	3.07 ± 1.34
DAS28-PCR	2.70 ± 1.05
SDAI	12 (7–19)
CDAI	8 (4–14)
History of extraarticular manifestations, *n* (%)	30 (11)
Erosions, *n* (%)	135 (44)
Current drugs, *n* (%)	
Prednisone	120 (36)
Prednisone doses, mg/day	5 (3–5)
NSAIDs	160 (47)
DMARDs	288 (85)
Methotrexate	245 (72)
Leflunomide	72 (21)
Hydroxychloroquine	37 (11)
Salazopyrin	24 (7)
Anti TNF therapy	68 (20)
Tocilizumab	22 (7)
Rituximab	7 (2)
Abatacept	10 (3)
Baricitinib	5 (1)
Tofacitinib	10 (3)

Data represent mean ± SD or median (IQR) when data were not normally distributed

*CV*, cardiovascular; *LDL*, low-density lipoprotein; *HDL*, high-density lipoprotein; *CRP*, C reactive protein; *NSAID*, Nonsteroidal anti-inflammatory drugs; *DMARD*, disease-modifying antirheumatic drug; *TNF*, tumor necrosis factor; Obesity; *ESR*, erythrocyte sedimentation rate; *BMI*, body mass index; *DAS28*, Disease Activity Score in 28 joints; *ACPA*, anti-citrullinated protein antibodies; *CDAI*, Clinical Disease Activity Index; *SDAI*, Simple Disease Activity Index; *SDAI*, Simple Disease Activity Index

The median duration of the disease was 8 (IQR 4–15) years. Seventy-five percent of patients were positive for rheumatoid factor, and 62% for anti-citrullinated protein antibodies (ACPA). Disease activity measured by DAS28-ESR yielded a value of 3.07 ± 1.34. Thirty-six percent of the patients were being treated with prednisone and 85% were taking at least one conventional DMARD, being methotrexate the most widely used (72%). The frequency of use of other treatments is shown in Table 1. Additionally, the mean values of CRP and ESR at the time of the study were 2.6 (IQR 1.2–5.6) mg/l and 18 (IQR 7–32) mm/1st hour, respectively. Further information on the patients is shown in Table 1.

### Relationship of demographics, cardiovascular risk factors, and disease characteristics with apolipoprotein C3

Neither age, sex, CV risk factors, nor statin use were associated with ApoC3. Only abdominal circumference showed a significant and positive relationship with circulating ApoC3 (beta coefficient (beta coef.) 0.07 [95%CI (confidence interval) 0.03–0.11] mg/dl, *p* = 0.001) (Table 2). Similarly, lipid pattern molecules were not related to ApoC3 with the exception of triglycerides that disclosed a positive relationship with higher serum levels of ApoC3. Concerning disease-related data, CRP (beta coef. 0.04 [95%CI 0.01–0.08] mg/dl, *p* = 0.021) and ESR (beta coef. 0.03 [95%CI 0.00–0.06] mg/dl, *p* = 0.021) showed a positive and significant relationship with circulating ApoC3; and disease activity through DAS28-ESR also showed a significant association with higher ApoC3 serum levels (beta coef. 0.55 [95%CI 0.18–0.93], *p* = 0.004). Remarkably, the use of leflunomide, salazopyrin and hydroxychloroquine was associated with lower ApoC3 levels. A similar trend was observed for the intake of methotrexate but in this case, the statistical significance was not achieved. Besides, prednisone dose –mg/day– was associated with lower levels of ApoC3 (beta coef. − 0.33 [95%CI − 0.54 to − 0.11] mg/dl, *p* = 0.004). Additional information regarding the relationship with disease-related data is shown in Table 2.

**Table 2:** Demographics, cardiovascular risk factors, and disease characteristics relationship with apolipoprotein C-III

	Apolipoprotein C-III, mg/dl	
	Beta coef. (95%CI)	
Age, years	− 0.03 (− 0.08–0.03)	0.35
Women, *n* (%)	− 0.82 (− 2.20–0.56)	0.25
BMI, kg/m^2^	0.00 (− 0.03–0.03)	0.89
Abdominal circumference, cm	**0.07 (0.03–0.11)**	**0.001**
Cardiovascular data		
CV risk factors, *n* (%)		
Current smoker	− 1.11 (− 2.34–0.13)	0.079
Obesity	0.98 (− 0.14–2.10)	0.087
Hypertension	− 0.26 (− 1.39–0.86)	0.64
Diabetes mellitus	–	
Statins, *n* (%)	0.39 (− 0.74–1.53)	0.50
Lipids		
Total cholesterol, mg/dl	0.00 (− 0.01–0.02)	0.75
Triglycerides, mg/dl	**0.01 (0.00–0.02)**	**0.001**
HDL cholesterol, mg/dl	0.03 (− 0.06–0.01)	0.15
LDL cholesterol, mg/dl	0.00 (− 0.02–0.01)	0.57
LDL:HDL cholesterol ratio	0.21 (− 0.36–0.77)	0.47
Non-HDL cholesterol, mg/dl	0.01 (− 0.01–0.02)	0.38
Lipoprotein (a), mg/dl	0.00 (− 0.01–0.01)	0.76
Apolipoprotein A1, mg/dl	− 0.01 (− 0.02–0.01)	0.42
Apolipoprotein B, mg/dl	0.00 (− 0.01–0.01)	0.66
Apo B:Apo A ratio	0.12 (− 1.97–2.20)	0.91
Disease-related data		
Disease duration, years	− **0.06 (**− **0.11–0.00)**	**0.048**
CRP at time of study, mg/l	**0.04 (0.01–0.08)**	**0.021**
ESR at time of study, mm/ 1º h	**0.03 (0.00–0.06)**	**0.021**
Rheumatoid factor, *n* (%)	− 0.34 (− 1.54–0.86)	0.58
ACPA, *n* (%)	0.56 (− 0.52–1.64)	0.31
DAS28-ESR	**0.55 (0.18–0.93)**	**0.004**
DAS28-PCR	0.33 (− 0.15–0.82)	0.18
SDAI	0.02 (− 0.02–0.07)	0.34
CDAI	0.01 (− 0.06–0.08)	0.79
History of extraarticular manifestations, *n* (%)	− 0.76 (− 2.61–1.10)	0.42
Erosions, *n* (%)	− **1.59 (–2.67 to** − **0.52)**	**0.004**
Current drugs, *n* (%)		
Prednisone	− 0.05 (− 1.13–1.03)	0.93
Prednisone doses, mg/day	− **0.33 (**− **0.54 to** − **0.11)**	**0.004**
NSAIDs	− 0.28 (− 1.31–0.75)	0.59
DMARDs	− 1.35 (− 2.81–0.11)	0.071
Methotrexate	− 0.92 (− 2.07–0.23)	0.12
Leflunomide	− **2.21 (**− **3.43 to** − **0.98)**	** < 0.001**
Hydroxychloroquine	− **4.33 (**− **5.89 to** − **2.78)**	** < 0.001**
Salazopyrin	− **4.32 (**− **6.24 to** − **2.41)**	** < 0.001**
Anti TNF therapy	− 0.79 (− 2.05–0.47)	0.22
Tocilizumab	− 0.34 (− 2.40–1.71)	0.74
Rituximab	1.88 (− 1.67–5.43)	0.30
Abatacept	1.12 (− 1.86–4.11)	0.46
Baricitinib	− 1.73 (− 5.92–2.46)	0.42
Tofacitinib	− **3.48 (**− **6.48–0.52)**	**0.022**

Beta coefficients consider ApoC3 as the dependent variable in this analysis. Significant *p* values are depicted in bold

*CV*, cardiovascular; *LDL*, low-density lipoprotein; *HDL*, high-density lipoprotein; *CRP*, C reactive protein; *NSAID*, nonsteroidal anti-inflammatory drugs; *DMARD*, disease-modifying antirheumatic drug; *TNF*, tumor necrosis factor; Obesity; *ESR*, erythrocyte sedimentation rate; *BMI*, body mass index; *DAS28*, Disease Activity Score in 28 joints; *ACPA*, anti-citrullinated protein antibodies; *CDAI*, Clinical Disease Activity Index; *SDAI*, Simple Disease Activity Index; *SDAI*, Simple Disease Activity Index

### Association between ApoC3 and glucose HOMA2 indices in patients with rheumatoid arthritis

Insulin and C-peptide serum levels and all HOMA2 indices, with the exception of HOMA2-%S, significantly correlated with ApoC3 in the univariable analysis (Table 3). After fully multivariable analysis, that included traditional factors associated with IR (abdominal circumference, smoking, obesity and triglycerides) and disease-related data (disease duration and activity, and prednisone and DMARDs intake) ApoC3, as independent variable, maintained similar significant relationships. In this sense, ApoC3 was associated with higher circulating insulin (beta coef. 0.37 [95%CI 0.01–0.73] µU/ml, *p* = 0.044) and C-peptide (beta coef. 0.13 [95%CI 0.05–0.22] ng/ml, *p* = 0.003). Similarly, ApoC3 was associated with significantly higher HOMA2-IR indices (both when this index was constructed with insulin or with C-peptide). Remarkably, ApoC3 also showed a positive relationship with beta-cell function HOMA2-%B-C-peptide index (beta coef. 2.94 [95%CI 0.07–5.80], *p* = 0.045) (Table 3).

**Table 3:** Association between apolipoprotein C3 and glucose homeostasis molecules and indices in patients with RA

		Apolipoprotein C-III, mg/dl					
		Pearson’s *r*	*p*	Beta coef. (95% CI)			
				Model 1	*p*	Model 2	*p*
Glucose, mg/dl	87 ± 10	− 0.050	0.37	− 0.22 (− 0.45–0.02)	0.076	0.16 (− 0.34–0.66)	0.52
Insulin, µU/ml	7.7 (5.2–12.2)	**0.199**	** < 0.001**	0.16 (− 0.02–0.34)	0.086	**0.37 (0.01–0.73)**	**0.044**
C-peptide, ng/ml	2.4 (1.5–3.4)	**0.234**	** < 0.001**	**0.06 (0.01–0.10)**	**0.011**	**0.13 (0.05–0.22)**	**0.003**
HOMA2-IR	1.00 (0.66–1.56)	**0.199**	** < 0.001**	0.02 (0.00–0.04)	0.088	**0.05 (0.00–0.09)**	**0.041**
HOMA2-%S	101 (64–151)	− 0.088	0.15	0.60 (− 1.03–2.22)	0.47		
HOMA2-%B	101 (80–137)	**0.199**	** < 0.001**	1.26 (− 0.04–2.55)	0.058	0.98 (− 0.40–2.36)	0.16
HOMA2-IR-C-peptide	1.66 (1.09–2.48)	**0.232**	** < 0.001**	**0.04 (0.01–0.07)**	**0.012**	**0.10 (0.04–0.17)**	**0.003**
HOMA2-%S-C-peptide	60 (40–92)	− **0.154**	**0.005**	− 0.26 (− 1.10–0.59)	0.55		
HOMA2-%B-C-peptide	145 (115–192)	**0.236**	** < 0.001**	**2.23 (0.64–3.82)**	**0.006**	**2.94 (0.07–5.80)**	**0.045**

Data represent means ± SD or median (IQR) when data were not normally distributed

In the multivariable analysis ApoC3 is considered the independent variable. Significant *p* values are depicted in bold

*HOMA2-IR*, Homeostatic Assessment Model for the assessment of insulin resistance using insulin and glucose serum levels; *HOMA2-%B-C peptide*, Homeostatic Assessment Model for the assessment of beta-cell function using C-peptide and glucose serum levels; *Model 1*, Adjusted for abdominal circumference, smoking, obesity, and triglycerides; *Model 2*, Adjusted for model 1 + disease duration, DAS28-ESR, prednisone dose and conventional DMARD use

## Discussion

In the present work, it is demonstrated, for the first time, that ApoC3 can be a molecule involved in the development of IR and beta-cell dysfunction in patients with RA. According to our results, both ApoC3 and IR are independently related, which would indicate that ApoC3 is involved in the metabolic pathways that lead to the alteration of carbohydrate metabolism that occurs in patients with RA.

Although IR states have been widely described in patients with RA, in our study IR index was not elevated. That is, the HOMA2-IR value in our population was around the unity (a HOMA2-IR of 1 is considered normal). IR has been shown to correlate with disease activity and inflammatory markers. For this reason, the fact that most patients in our series had moderate disease activity can justify this finding. This is relevant since despite the fact that patients with RA did not show high IR indexes, we were capable to describe a significant relationship between these IR and beta-cell dysfunction indices and ApoC3.

ApoC3 has deserved prior attention in RA. For example, in a previous study of 152 RA patients in whom coronary artery calcium was assessed at baseline and at year 3, ApoC3 was found to be significantly elevated in those in whom coronary artery calcium worsened compared to non-progressors [19]. In that study, ApoC3 was considered a risk factor for the progression of atherosclerosis in patients with RA. In another report, baricitinib-associated increment in serum lipid levels from baseline to week 12 included the upregulation of ApoC3 [20]. Furthermore, after six months of treatment with etanercept, ApoC3 showed significantly higher levels in non-responders compared to those who met the criteria for improvement [21]. However, until our current work, previous studies have not evaluated the role of ApoC3 in the IR of patients with RA.

Triglycerides have been shown to correlate with both IR and beta cell function in the general population [22]. Moreover, ApoC3 is a key regulator of triglyceride metabolism through its inhibition of lipoprotein lipase, which, in turn, degrades circulating triglycerides. In our study, in patients with rheumatoid arthritis, circulating ApoC3 was also significantly correlated with serum triglyceride levels. However, the association of ApoC3 with IR and beta-cell dysfunction found in our study cannot be attributed to its relationship with triglycerides, as it remained significant after adjusting for triglycerides. For this reason, we believe that the association between ApoC3 and IR and beta cell function in patients with RA is direct in nature and not related to triglycerides.

Interestingly, in our work the acute phase reactants, CRP and ESR, as well as the clinical disease activity scores, showed a positive and significant relationship with elevated ApoC3 levels. This relationship between ApoC3 and inflammation has been previously described. In this sense, in a former study, ApoC3 was identified as a novel mediator leading to alternative inflammasome activation in human monocytes [23]. Besides, although dyslipidemia is a known adverse effect of tocilizumab we did not find a relation between this drug and ApoC3 serum levels. We understand that the cross-sectional design of our study and the small number of patients receiving this drug motivate to take this result with caution.

Our work may have some clinical implications. Since ApoC3 and IR have been both related to CV disease in the general population, and they seem to be linked processes, we believe the assessment of serum levels of ApoC3 may serve as a predictor of CV disease or as an indirect marker of the presence of IR in patients with RA.

The purpose of our study focused specifically on patients with RA, the prototype of chronic inflammatory disease. For this reason, we did not include a control group of healthy individuals. It could be considered as a potential limitation of our study. We also acknowledge that this was a cross-sectional study and, hence, inferences on the direction of causality cannot be made.

## Conclusion

In conclusion, ApoC3, insulin resistance, and beta-cell dysfunction are independently associated in patients with RA. We believe our findings expand to ApoC3 the understanding of the signaling pathways necessary for the development of IR and beta-cell dysfunction in this population.

## Data Availability

The data sets used and/or analyzed in the present study are available from the corresponding author upon request.

## Abbreviations

ACPA: Anti-citrullinated protein antibodies
ApoC3: Apolipoprotein C-III
BMI: Body mass index
CDAI: Clinical Disease Activity Index
CI: Confidence interval
CRP: C reactive protein
CV: Cardiovascular
DAS28: Disease Activity Score in 28 joints
DMARD: Disease-modifying antirheumatic drug
ELISA: Enzyme-linked immunosorbent assay
ESR: Erythrocyte sedimentation rate
HDL: High-density lipoprotein
HOMA: Homeostasis Model Assessment
LDL: Low-density lipoprotein
IQR: Interquartile range
IR: Insulin resistance
NSAID: Nonsteroidal anti-inflammatory drugs
TNF: Tumor necrosis factor
RA: Rheumatoid arthritis
SD: Standard deviation
SDAI: Simple Disease Activity Index
